# (2,4-Dioxo-1,2,3,4-tetra­hydro­pyrimi­dine-5-carboxyl­ato-κ^2^ *O* ^4^,*O* ^5^)(4-oxido-2-oxo-1,2-dihydro­pyrimidine-5-carboxyl­ato-κ^2^ *O* ^4^,*O* ^5^)bis­(1,10-phenanthroline-κ^2^ *N*,*N*′)yttrium(III) dihydrate

**DOI:** 10.1107/S1600536808025701

**Published:** 2008-08-16

**Authors:** Wei Xiong, Huihui Xing, Yan Su, Zilu Chen

**Affiliations:** a College of Chemistry and Chemical Engineering, Guangxi Normal University, Yucai Road 15, Guilin 541004, People’s Republic of China

## Abstract

In the title compound, [Y(C_5_H_2_N_2_O_4_)(C_5_H_3_N_2_O_4_)(C_12_H_8_N_2_)_2_]·2H_2_O, the Y^III^ ion lies on a twofold rotation axis and exhibits a distorted square-anti­prismatic coordination geometry. It is chelated by two 1,10-phenanthroline ligands, a 2,4-dioxo-1,2,3,4-tetra­hydro­pyrimidine-5-carboxyl­ate mono­anion and a 4-oxido-2-oxo-1,2-dihydro­pyrimidine-5-carboxyl­ate dianion. The H atom involved in an N—H⋯N hydrogen bond between the 1,2-dihydro­pyrimidine units has half occupancy and is disordered around a twofold rotation axis.

## Related literature

For the crystal structures of the isostructural Er, Eu, Tb and Yb complexes, see: Sun & Jin (2004 ▶); Xing *et al.* (2008 ▶). For other related literature, see: Tobiki *et al.* (1980 ▶); Castan *et al.* (1990 ▶).
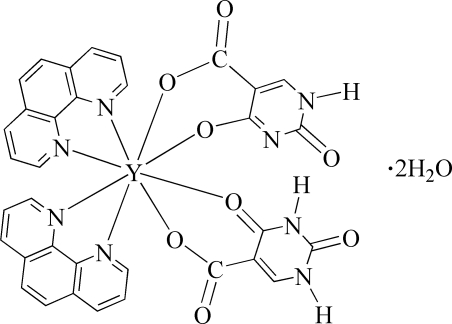


## Experimental

### 

#### Crystal data

[Y(C_5_H_2_N_2_O_4_)(C_5_H_3_N_2_O_4_)(C_12_H_8_N_2_)_2_]·2H_2_O
*M* *_r_* = 794.53
Monoclinic, 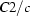

*a* = 17.1740 (13) Å
*b* = 14.4385 (11) Å
*c* = 13.2365 (10) Å
β = 100.881 (1)°
*V* = 3223.2 (4) Å^3^
*Z* = 4
Mo *K*α radiation
μ = 1.89 mm^−1^
*T* = 295 (2) K
0.24 × 0.08 × 0.06 mm

#### Data collection

Bruker APEXII diffractometer
Absorption correction: multi-scan (*SADABS*; Bruker, 1998 ▶) *T*_min_ = 0.660, *T*_max_ = 0.895
12035 measured reflections
3152 independent reflections
2986 reflections with *I* > 2σ(*I*)
*R*_int_ = 0.035

#### Refinement

*R*[*F* ^2^ > 2σ(*F* ^2^)] = 0.045
*wR*(*F* ^2^) = 0.096
*S* = 1.21
3152 reflections
240 parameters
H-atom parameters constrained
Δρ_max_ = 0.41 e Å^−3^
Δρ_min_ = −0.51 e Å^−3^

### 

Data collection: *APEX2* (Bruker, 2004 ▶); cell refinement: *SAINT* (Bruker, 2004 ▶); data reduction: *SAINT*; program(s) used to solve structure: *SHELXS97* (Sheldrick, 2008 ▶); program(s) used to refine structure: *SHELXL97* (Sheldrick, 2008 ▶); molecular graphics: *SHELXTL* (Sheldrick, 2008 ▶); software used to prepare material for publication: *SHELXTL*.

## Supplementary Material

Crystal structure: contains datablocks global, I. DOI: 10.1107/S1600536808025701/gk2161sup1.cif

Structure factors: contains datablocks I. DOI: 10.1107/S1600536808025701/gk2161Isup2.hkl

Additional supplementary materials:  crystallographic information; 3D view; checkCIF report

## Figures and Tables

**Table:** 

Y1—O2	2.247 (2)
Y1—O1	2.302 (2)
Y1—N1	2.547 (3)
Y1—N2	2.573 (3)

**Table:** 

O2^i^—Y1—O2	89.15 (13)
O2—Y1—O1^i^	81.79 (9)
O2—Y1—O1	74.69 (8)
O1^i^—Y1—O1	146.81 (11)
O2^i^—Y1—N1	147.62 (8)
O2—Y1—N1	105.31 (9)
O1^i^—Y1—N1	135.17 (8)
O1—Y1—N1	74.65 (9)
N1—Y1—N1^i^	77.82 (13)
O2—Y1—N2^i^	148.38 (8)
O1—Y1—N2^i^	74.55 (8)
N1—Y1—N2^i^	73.15 (9)
O2—Y1—N2	79.43 (9)
O1—Y1—N2	122.30 (9)
N1—Y1—N2	63.90 (9)
N2^i^—Y1—N2	124.07 (12)

Symmetry code: (i) 
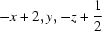
.

**Table 2:** Hydrogen-bond geometry (Å, °)

*D*—H⋯*A*	*D*—H	H⋯*A*	*D*⋯*A*	*D*—H⋯*A*
N1—H1⋯O2^ii^	0.86	2.04	2.898 (3)	178
N1—H1⋯O1^ii^	0.86	2.60	3.160 (4)	124
N2—H2⋯N2^iii^	0.86	1.81	2.669 (5)	174
O5—H5*A*⋯O4^iv^	0.85	2.14	2.970 (4)	164
O5—H5*B*⋯O2^v^	0.85	2.14	2.985 (4)	173

Symmetry codes: (ii) 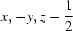; (iii) 
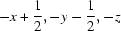
; (iv) 
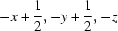
; (v) 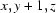.

**Fig. 1.**
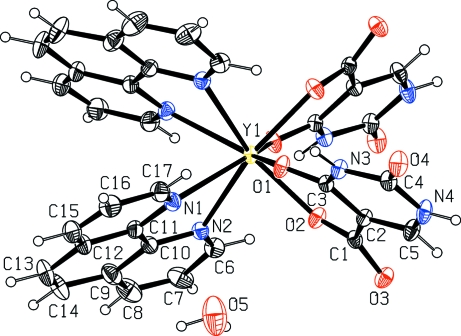
The molecular structure of the title compound with the atom-numbering scheme and 30% displacement ellipsoids.

## supplementary crystallographic information

### Comment

2,4-Dioxo-1,2,3,4-tetrahydropyrimidine-5-carboxylic acid has attracted much attention because of its potential biological activity and pharmaceutical properties, such as anticancer, antibacterial and antihypertensive properties (Tobiki *et al.*, 1980; Castan *et al.*, 1990). Here we report the crystal structure of Y^III^ complex with 2,4-dioxo-1,2,3,4-tetrahydropyrimidine-5-carboxylic acid and 1,10-phenenthroline as ligands. The title complex turned out to be isostructural with the analogues Eu, Tb, Yb (Sun & Jin, 2004) and Er (Xing *et al.*, 2008) complexes; see Sun & Jin (2004) for their detailed description.

### Experimental

A mixture of 2,4-dihydroxypyrimidine-5-carboxylic acid (0.0312 g, 0.2 mmol), YCl_3_.6H_2_O (0.0607 g, 0.2 mmol), phenanthroline dihydrate (0.0396 g, 0.2 mmol), NaOH (0.0160 g, 0.4 mmol) and water (15 ml) was sealed in a 25 ml, Teflon-lined stainless-steel reactor and heated to 383 K for 120 h. It was then cooled over 48 h to give colourless crystals in 70% yield. Elemental analysis calculated for C_34_H_25_N_8_O_10_Y: C 51.40, H 3.17, N 14.10%; found: C 51.77, H 3.29, N 14.49%.

### Refinement

H atoms of the water molecule were located in a difference Fourier map and allowed to ride on their parent atom [*U*_iso_(H) = 1.5*U*_eq_(O)]. Other H atoms were placed at calculated positions (C—H = 0.93 Å and N—H = 0.86 Å) and were included in the refinement in the riding model approximation, with *U*_iso_(H) = 1.2*U*_eq_(C, N). The pyrimidine hydrogen atom H4 is shared by two N—H groups and thus has an occupancy factor of 0.5.

### Crystal data

**Table:**

[Y(C_5_H_2_N_2_O_4_)(C_5_H_3_N_2_O_4_)(C_12_H_8_N_2_)_2_]·2H_2_O	*F*_000_ = 1616
*M**_r_* = 794.53	*D*_x_ = 1.637 Mg m^−^^3^
Monoclinic, *C*2/*c*	Mo *K*α radiation λ = 0.71073 Å
Hall symbol: -C 2yc	Cell parameters from 7855 reflections
*a* = 17.1740 (13) Å	θ = 2.3–27.9º
*b* = 14.4385 (11) Å	µ = 1.89 mm^−^^1^
*c* = 13.2365 (10) Å	*T* = 295 (2) K
β = 100.8810 (10)º	Prism, colourless
*V* = 3223.2 (4) Å^3^	0.24 × 0.08 × 0.06 mm
*Z* = 4	

### Data collection

**Table:**

Bruker APEXII diffractometer	3152 independent reflections
Radiation source: fine-focus sealed tube	2986 reflections with *I* > 2σ(*I*)
Monochromator: graphite	*R*_int_ = 0.035
*T* = 295(2) K	θ_max_ = 26.0º
φ and ω scans	θ_min_ = 1.9º
Absorption correction: multi-scan(SADABS; Bruker, 1998)	*h* = −21→21
*T*_min_ = 0.660, *T*_max_ = 0.895	*k* = −17→17
12035 measured reflections	*l* = −16→16

### Refinement

**Table:**

Refinement on *F*^2^	Secondary atom site location: difference Fourier map
Least-squares matrix: full	Hydrogen site location: inferred from neighbouring sites
*R*[*F*^2^ > 2σ(*F*^2^)] = 0.046	H-atom parameters constrained
*wR*(*F*^2^) = 0.096	*w* = 1/[σ^2^(*F*_o_^2^) + (0.0141*P*)^2^ + 9.88*P*] where *P* = (*F*_o_^2^ + 2*F*_c_^2^)/3
*S* = 1.21	(Δ/σ)_max_ < 0.001
3152 reflections	Δρ_max_ = 0.41 e Å^−^^3^
240 parameters	Δρ_min_ = −0.51 e Å^−^^3^
Primary atom site location: structure-invariant direct methods	Extinction correction: none

### Special details

**Table:**

Geometry. All esds (except the esd in the dihedral angle between two l.s. planes) are estimated using the full covariance matrix. The cell esds are taken into account individually in the estimation of esds in distances, angles and torsion angles; correlations between esds in cell parameters are only used when they are defined by crystal symmetry. An approximate (isotropic) treatment of cell esds is used for estimating esds involving l.s. planes.
Refinement. Refinement of F^2^ against ALL reflections. The weighted R-factor wR and goodness of fit S are based on F^2^, conventional R-factors R are based on F, with F set to zero for negative F^2^. The threshold expression of F^2^ > 2sigma(F^2^) is used only for calculating R-factors(gt) etc. and is not relevant to the choice of reflections for refinement. R-factors based on F^2^ are statistically about twice as large as those based on F, and R- factors based on ALL data will be even larger.

### Fractional atomic coordinates and isotropic or equivalent isotropic displacement parameters (Å^2^)

**Table:**

	*x*	*y*	*z*	*U*_iso_*/*U*_eq_	Occ. (<1)
Y1	1.0000	0.88802 (3)	0.2500	0.02339 (12)	
N1	0.90591 (16)	0.75076 (19)	0.2117 (2)	0.0324 (6)	
N2	0.96510 (16)	0.80446 (19)	0.4073 (2)	0.0304 (6)	
N3	0.83326 (16)	1.02589 (19)	−0.0065 (2)	0.0297 (6)	
H3	0.8379	0.9842	−0.0514	0.036*	
N4	0.78240 (19)	1.1684 (2)	0.0310 (2)	0.0387 (7)	
H4	0.7585	1.2199	0.0131	0.046*	0.50
O1	0.90470 (14)	0.93356 (15)	0.11247 (17)	0.0329 (6)	
O2	0.93006 (14)	0.99886 (16)	0.31195 (17)	0.0337 (6)	
O3	0.84729 (15)	1.10907 (17)	0.34049 (17)	0.0371 (6)	
O4	0.76917 (16)	1.11986 (18)	−0.13684 (19)	0.0451 (7)	
C1	0.8770 (2)	1.0594 (2)	0.2805 (2)	0.0272 (7)	
C2	0.84980 (19)	1.0723 (2)	0.1678 (2)	0.0269 (7)	
C3	0.86584 (18)	1.0066 (2)	0.0941 (2)	0.0248 (7)	
C4	0.7937 (2)	1.1063 (2)	−0.0423 (3)	0.0324 (7)	
C5	0.8085 (2)	1.1498 (2)	0.1311 (3)	0.0358 (8)	
H5	0.7976	1.1928	0.1788	0.043*	
C6	0.9900 (2)	0.8317 (3)	0.5036 (3)	0.0386 (8)	
H6	1.0089	0.8920	0.5148	0.046*	
C7	0.9896 (3)	0.7758 (3)	0.5890 (3)	0.0512 (11)	
H7	1.0074	0.7982	0.6552	0.061*	
C8	0.9624 (3)	0.6872 (3)	0.5735 (3)	0.0599 (12)	
H8	0.9626	0.6483	0.6296	0.072*	
C9	0.9342 (2)	0.6545 (3)	0.4735 (3)	0.0463 (10)	
C10	0.9355 (2)	0.7169 (2)	0.3922 (3)	0.0327 (8)	
C11	0.9042 (2)	0.6886 (2)	0.2883 (3)	0.0325 (8)	
C12	0.8716 (2)	0.5993 (2)	0.2699 (3)	0.0419 (9)	
C13	0.8735 (3)	0.5372 (3)	0.3546 (4)	0.0607 (13)	
H13	0.8534	0.4776	0.3423	0.073*	
C14	0.9036 (3)	0.5634 (3)	0.4513 (4)	0.0618 (13)	
H14	0.9045	0.5215	0.5048	0.074*	
C15	0.8370 (2)	0.5768 (3)	0.1686 (4)	0.0510 (11)	
H15	0.8148	0.5185	0.1533	0.061*	
C16	0.8357 (2)	0.6401 (3)	0.0926 (3)	0.0490 (10)	
H16	0.8119	0.6262	0.0252	0.059*	
C17	0.8707 (2)	0.7264 (3)	0.1173 (3)	0.0397 (9)	
H17	0.8693	0.7693	0.0647	0.048*	
O5	0.6925 (3)	0.1887 (2)	0.6600 (3)	0.0956 (14)	
H51	0.6793	0.2454	0.6644	0.143*	
H52	0.7082	0.1681	0.7208	0.143*	

### Atomic displacement parameters (Å^2^)

**Table:**

	*U*^11^	*U*^22^	*U*^33^	*U*^12^	*U*^13^	*U*^23^
Y1	0.0287 (2)	0.0187 (2)	0.0207 (2)	0.000	−0.00062 (16)	0.000
N1	0.0322 (16)	0.0288 (15)	0.0352 (16)	−0.0037 (12)	0.0040 (12)	−0.0035 (12)
N2	0.0318 (15)	0.0294 (15)	0.0297 (15)	−0.0002 (12)	0.0051 (12)	0.0008 (12)
N3	0.0389 (16)	0.0276 (15)	0.0204 (13)	0.0051 (12)	0.0001 (12)	−0.0021 (11)
N4	0.057 (2)	0.0273 (15)	0.0278 (15)	0.0107 (14)	−0.0016 (14)	0.0018 (12)
O1	0.0421 (14)	0.0265 (12)	0.0255 (12)	0.0100 (10)	−0.0052 (10)	−0.0034 (9)
O2	0.0441 (14)	0.0324 (13)	0.0226 (12)	0.0133 (11)	0.0011 (10)	0.0006 (10)
O3	0.0510 (15)	0.0374 (14)	0.0227 (12)	0.0125 (12)	0.0064 (11)	−0.0021 (10)
O4	0.0562 (17)	0.0369 (15)	0.0358 (14)	0.0044 (13)	−0.0074 (12)	0.0002 (12)
C1	0.0345 (18)	0.0240 (16)	0.0224 (16)	−0.0025 (14)	0.0032 (13)	0.0017 (13)
C2	0.0328 (17)	0.0263 (16)	0.0207 (16)	0.0053 (14)	0.0031 (13)	0.0002 (13)
C3	0.0284 (16)	0.0237 (16)	0.0209 (15)	−0.0008 (13)	0.0014 (13)	0.0007 (12)
C4	0.0373 (19)	0.0291 (18)	0.0280 (17)	−0.0006 (15)	−0.0012 (14)	0.0021 (14)
C5	0.050 (2)	0.0292 (18)	0.0263 (18)	0.0094 (16)	0.0031 (16)	−0.0026 (14)
C6	0.039 (2)	0.042 (2)	0.0339 (19)	−0.0026 (17)	0.0042 (16)	−0.0029 (16)
C7	0.056 (3)	0.070 (3)	0.028 (2)	−0.010 (2)	0.0082 (18)	0.0061 (19)
C8	0.066 (3)	0.069 (3)	0.044 (2)	−0.008 (2)	0.009 (2)	0.024 (2)
C9	0.048 (2)	0.043 (2)	0.048 (2)	−0.0034 (19)	0.0101 (19)	0.0158 (19)
C10	0.0306 (18)	0.0296 (18)	0.0384 (19)	−0.0007 (14)	0.0078 (15)	0.0034 (15)
C11	0.0311 (18)	0.0269 (17)	0.0399 (19)	−0.0005 (14)	0.0072 (15)	−0.0017 (15)
C12	0.042 (2)	0.0279 (19)	0.058 (2)	−0.0074 (16)	0.0137 (19)	−0.0027 (17)
C13	0.074 (3)	0.030 (2)	0.080 (3)	−0.018 (2)	0.017 (3)	0.004 (2)
C14	0.076 (3)	0.042 (3)	0.068 (3)	−0.017 (2)	0.016 (3)	0.021 (2)
C15	0.051 (2)	0.035 (2)	0.069 (3)	−0.0161 (19)	0.016 (2)	−0.018 (2)
C16	0.047 (2)	0.050 (2)	0.049 (2)	−0.0137 (19)	0.0063 (19)	−0.022 (2)
C17	0.039 (2)	0.039 (2)	0.040 (2)	−0.0064 (17)	0.0052 (16)	−0.0062 (16)
O5	0.145 (4)	0.059 (2)	0.067 (2)	0.004 (2)	−0.017 (2)	−0.0089 (19)

### Geometric parameters (Å, °)

**Table:**

Y1—O2^i^	2.247 (2)	C2—C3	1.423 (4)
Y1—O2	2.247 (2)	C5—H5	0.9300
Y1—O1^i^	2.302 (2)	C6—C7	1.391 (5)
Y1—O1	2.302 (2)	C6—H6	0.9300
Y1—N1	2.547 (3)	C7—C8	1.362 (6)
Y1—N1^i^	2.547 (3)	C7—H7	0.9300
Y1—N2^i^	2.573 (3)	C8—C9	1.403 (6)
Y1—N2	2.573 (3)	C8—H8	0.9300
N1—C17	1.329 (4)	C9—C10	1.407 (5)
N1—C11	1.358 (4)	C9—C14	1.426 (6)
N2—C6	1.326 (4)	C10—C11	1.439 (5)
N2—C10	1.363 (4)	C11—C12	1.408 (5)
N3—C3	1.373 (4)	C12—C15	1.399 (6)
N3—C4	1.383 (4)	C12—C13	1.431 (6)
N3—H3	0.8600	C13—C14	1.342 (6)
N4—C5	1.343 (4)	C13—H13	0.9300
N4—C4	1.362 (4)	C14—H14	0.9300
N4—H4	0.8600	C15—C16	1.355 (6)
O1—C3	1.247 (4)	C15—H15	0.9300
O2—C1	1.275 (4)	C16—C17	1.395 (5)
O3—C1	1.248 (4)	C16—H16	0.9300
O4—C4	1.258 (4)	C17—H17	0.9300
C1—C2	1.489 (4)	O5—H51	0.8533
C2—C5	1.364 (4)	O5—H52	0.8518
			
O2^i^—Y1—O2	89.15 (13)	C3—C2—C1	122.6 (3)
O2^i^—Y1—O1^i^	74.69 (8)	O1—C3—N3	117.7 (3)
O2—Y1—O1^i^	81.79 (9)	O1—C3—C2	126.6 (3)
O2^i^—Y1—O1	81.79 (8)	N3—C3—C2	115.7 (3)
O2—Y1—O1	74.69 (8)	O4—C4—N4	122.7 (3)
O1^i^—Y1—O1	146.81 (11)	O4—C4—N3	121.5 (3)
O2^i^—Y1—N1	147.62 (8)	N4—C4—N3	115.8 (3)
O2—Y1—N1	105.31 (9)	N4—C5—C2	124.8 (3)
O1^i^—Y1—N1	135.17 (8)	N4—C5—H5	117.6
O1—Y1—N1	74.65 (9)	C2—C5—H5	117.6
O2^i^—Y1—N1^i^	105.31 (9)	N2—C6—C7	124.0 (4)
O2—Y1—N1^i^	147.62 (8)	N2—C6—H6	118.0
O1^i^—Y1—N1^i^	74.65 (9)	C7—C6—H6	118.0
O1—Y1—N1^i^	135.17 (8)	C8—C7—C6	118.5 (4)
N1—Y1—N1^i^	77.82 (13)	C8—C7—H7	120.7
O2^i^—Y1—N2^i^	79.43 (8)	C6—C7—H7	120.7
O2—Y1—N2^i^	148.38 (8)	C7—C8—C9	120.4 (4)
O1^i^—Y1—N2^i^	122.30 (9)	C7—C8—H8	119.8
O1—Y1—N2^i^	74.55 (8)	C9—C8—H8	119.8
N1—Y1—N2^i^	73.15 (9)	C8—C9—C10	117.0 (4)
N1^i^—Y1—N2^i^	63.90 (9)	C8—C9—C14	123.6 (4)
O2^i^—Y1—N2	148.38 (8)	C10—C9—C14	119.4 (4)
O2—Y1—N2	79.43 (9)	N2—C10—C9	122.8 (3)
O1^i^—Y1—N2	74.55 (8)	N2—C10—C11	117.7 (3)
O1—Y1—N2	122.30 (9)	C9—C10—C11	119.5 (3)
N1—Y1—N2	63.90 (9)	N1—C11—C12	122.7 (3)
N1^i^—Y1—N2	73.15 (9)	N1—C11—C10	118.0 (3)
N2^i^—Y1—N2	124.07 (12)	C12—C11—C10	119.3 (3)
C17—N1—C11	117.1 (3)	C15—C12—C11	117.5 (4)
C17—N1—Y1	123.5 (2)	C15—C12—C13	123.2 (4)
C11—N1—Y1	117.9 (2)	C11—C12—C13	119.3 (4)
C6—N2—C10	117.4 (3)	C14—C13—C12	121.2 (4)
C6—N2—Y1	123.8 (2)	C14—C13—H13	119.4
C10—N2—Y1	117.0 (2)	C12—C13—H13	119.4
C3—N3—C4	125.9 (3)	C13—C14—C9	121.2 (4)
C3—N3—H3	117.0	C13—C14—H14	119.4
C4—N3—H3	117.0	C9—C14—H14	119.4
C5—N4—C4	120.3 (3)	C16—C15—C12	119.9 (4)
C5—N4—H4	119.9	C16—C15—H15	120.0
C4—N4—H4	119.9	C12—C15—H15	120.0
C3—O1—Y1	132.1 (2)	C15—C16—C17	118.8 (4)
C1—O2—Y1	140.2 (2)	C15—C16—H16	120.6
O3—C1—O2	122.6 (3)	C17—C16—H16	120.6
O3—C1—C2	118.5 (3)	N1—C17—C16	123.9 (4)
O2—C1—C2	118.8 (3)	N1—C17—H17	118.1
C5—C2—C3	117.2 (3)	C16—C17—H17	118.1
C5—C2—C1	120.2 (3)	H51—O5—H52	108.2
			
O2^i^—Y1—N1—C17	−8.9 (4)	C4—N3—C3—O1	−174.8 (3)
O2—Y1—N1—C17	104.9 (3)	C4—N3—C3—C2	6.6 (5)
O1^i^—Y1—N1—C17	−161.4 (3)	C5—C2—C3—O1	177.5 (3)
O1—Y1—N1—C17	35.9 (3)	C1—C2—C3—O1	−1.8 (5)
N1^i^—Y1—N1—C17	−108.3 (3)	C5—C2—C3—N3	−4.0 (5)
N2^i^—Y1—N1—C17	−42.2 (3)	C1—C2—C3—N3	176.7 (3)
N2—Y1—N1—C17	174.6 (3)	C5—N4—C4—O4	178.8 (4)
O2^i^—Y1—N1—C11	157.1 (2)	C5—N4—C4—N3	−0.5 (5)
O2—Y1—N1—C11	−89.1 (2)	C3—N3—C4—O4	176.4 (3)
O1^i^—Y1—N1—C11	4.6 (3)	C3—N3—C4—N4	−4.3 (5)
O1—Y1—N1—C11	−158.1 (3)	C4—N4—C5—C2	2.7 (6)
N1^i^—Y1—N1—C11	57.7 (2)	C3—C2—C5—N4	−0.3 (6)
N2^i^—Y1—N1—C11	123.8 (3)	C1—C2—C5—N4	179.0 (3)
N2—Y1—N1—C11	−19.3 (2)	C10—N2—C6—C7	1.8 (5)
O2^i^—Y1—N2—C6	7.0 (4)	Y1—N2—C6—C7	−162.4 (3)
O2—Y1—N2—C6	−63.6 (3)	N2—C6—C7—C8	0.4 (6)
O1^i^—Y1—N2—C6	20.7 (3)	C6—C7—C8—C9	−1.3 (7)
O1—Y1—N2—C6	−127.8 (3)	C7—C8—C9—C10	0.0 (7)
N1—Y1—N2—C6	−176.6 (3)	C7—C8—C9—C14	−178.8 (5)
N1^i^—Y1—N2—C6	99.0 (3)	C6—N2—C10—C9	−3.2 (5)
N2^i^—Y1—N2—C6	139.5 (3)	Y1—N2—C10—C9	162.2 (3)
O2^i^—Y1—N2—C10	−157.3 (2)	C6—N2—C10—C11	176.5 (3)
O2—Y1—N2—C10	132.1 (2)	Y1—N2—C10—C11	−18.1 (4)
O1^i^—Y1—N2—C10	−143.6 (2)	C8—C9—C10—N2	2.3 (6)
O1—Y1—N2—C10	67.9 (2)	C14—C9—C10—N2	−178.8 (4)
N1—Y1—N2—C10	19.1 (2)	C8—C9—C10—C11	−177.4 (4)
N1^i^—Y1—N2—C10	−65.3 (2)	C14—C9—C10—C11	1.5 (6)
N2^i^—Y1—N2—C10	−24.8 (2)	C17—N1—C11—C12	4.1 (5)
O2^i^—Y1—O1—C3	−68.1 (3)	Y1—N1—C11—C12	−162.8 (3)
O2—Y1—O1—C3	23.3 (3)	C17—N1—C11—C10	−174.4 (3)
O1^i^—Y1—O1—C3	−23.2 (3)	Y1—N1—C11—C10	18.7 (4)
N1—Y1—O1—C3	134.3 (3)	N2—C10—C11—N1	−0.2 (5)
N1^i^—Y1—O1—C3	−171.6 (3)	C9—C10—C11—N1	179.6 (3)
N2^i^—Y1—O1—C3	−149.3 (3)	N2—C10—C11—C12	−178.7 (3)
N2—Y1—O1—C3	89.8 (3)	C9—C10—C11—C12	1.1 (5)
O2^i^—Y1—O2—C1	72.0 (3)	N1—C11—C12—C15	−2.7 (6)
O1^i^—Y1—O2—C1	146.7 (4)	C10—C11—C12—C15	175.7 (3)
O1—Y1—O2—C1	−9.7 (3)	N1—C11—C12—C13	178.8 (4)
N1—Y1—O2—C1	−78.6 (3)	C10—C11—C12—C13	−2.8 (6)
N1^i^—Y1—O2—C1	−170.0 (3)	C15—C12—C13—C14	−176.4 (5)
N2^i^—Y1—O2—C1	4.0 (4)	C11—C12—C13—C14	2.0 (7)
N2—Y1—O2—C1	−137.6 (4)	C12—C13—C14—C9	0.6 (8)
Y1—O2—C1—O3	176.0 (2)	C8—C9—C14—C13	176.5 (5)
Y1—O2—C1—C2	−4.6 (5)	C10—C9—C14—C13	−2.3 (7)
O3—C1—C2—C5	15.4 (5)	C11—C12—C15—C16	−0.1 (6)
O2—C1—C2—C5	−164.0 (3)	C13—C12—C15—C16	178.4 (4)
O3—C1—C2—C3	−165.3 (3)	C12—C15—C16—C17	1.3 (6)
O2—C1—C2—C3	15.2 (5)	C11—N1—C17—C16	−2.8 (5)
Y1—O1—C3—N3	158.7 (2)	Y1—N1—C17—C16	163.3 (3)
Y1—O1—C3—C2	−22.8 (5)	C15—C16—C17—N1	0.2 (6)

Symmetry codes: (i) −*x*+2, *y*, −*z*+1/2.

### Hydrogen-bond geometry (Å, °)

**Table:**

*D*—H···*A*	*D*—H	H···*A*	*D*···*A*	*D*—H···*A*
N3—H3···O3^ii^	0.86	1.99	2.853 (4)	178
N3—H3···O2^ii^	0.86	2.63	3.189 (4)	124
N4—H4···N4^iii^	0.86	1.81	2.667 (6)	174
O5—H51···O3^iv^	0.85	2.15	2.998 (4)	173
O5—H52···O4^v^	0.85	2.10	2.935 (4)	169

Symmetry codes: (ii) *x*, −*y*+2, *z*−1/2; (iii) −*x*+3/2, −*y*+5/2, −*z*; (iv) −*x*+3/2, −*y*+3/2, −*z*+1; (v) *x*, *y*−1, *z*+1.

## References

[bb1] Bruker (1998). *SADABS* Bruker AXS Inc., Madison, Wisconsin, USA.

[bb2] Bruker (2004). *APEX2* and *SAINT* Bruker AXS Inc., Madison, Wisconsin, USA.

[bb3] Castan, P., Colacio-Rodriguez, E., Beauchamp, A. L., Cros, S. & Wimmer, J. (1990). *J. Inorg. Biochem.* **38**, 225–239. 10.1016/0162-0134(90)84015-h 2329345

[bb4] Sheldrick, G. M. (2008). *Acta Cryst.* A**64**, 112–122. 10.1107/S0108767307043930 18156677

[bb5] Sun, C.-Y. & Jin, L.-P. (2004). *Polyhedron*, **23**, 2227–2233.

[bb6] Tobiki, H., Yamada, H., Nakatsaka, I., Shimago, K., Eda, Y., Noguchi, H., Komatsu, T. & Nakagome, T. (1980). *Yakugaku Zasshi*, **100**, 38–48. 6900065

[bb7] Xing, H.-H., Chen, Z.-L. & Ng, S. W. (2008). *Acta Cryst.* E**64**, m418. 10.1107/S1600536808001487 PMC2960368 21201363

